# Out-of-Pocket Health Expenditure Among Migrant Workers in India: A Narrative Review

**DOI:** 10.7759/cureus.30948

**Published:** 2022-10-31

**Authors:** Sudha R Bhoi, Shiv H Joshi, Abhishek Joshi

**Affiliations:** 1 Epidemiology and Public Health, Jawaharlal Nehru Medical College, Datta Meghe Institute of Medical Sciences, Wardha, IND; 2 Community Medicine, Jawaharlal Nehru Medical College, Datta Meghe Institute of Medical Sciences, Wardha, IND

**Keywords:** out of pocket payments, health expenditure, migrant labour, out-of-pocket expenditure, migrant workers

## Abstract

Migrant workers make significant contributions to host regions and economies in many nations, frequently working in vulnerable situations in crucial sectors like agricultural and food processing, transportation, health care, and construction sectors. India has one of the world's highest out-of-pocket spending rates, with a cost making up about 62.6% of total health expenses. Migrant workers face healthcare financing burdens due to medical expenses resulting in large out-of-pocket payments. This narrative review aims to gain insight into why migrant workers face out-of-pocket expenditures on health and explore the inclusion of migrant workers in the existing healthcare system in India. For the literature search, databases like PubMed and Google Scholar were used to find relevant articles. This review will be helpful from the public health perspective in illustrating the need for studies and research on the health rights of migrant workers, their healthcare finances, and the social protection of such vulnerable populations who are poor and marginalized. Due to various health disparities, migrant workers may incur unforeseen out-of-pocket costs for the household healthcare system. Health insurance enhances the likelihood of accessing healthcare and minimizes out-of-pocket expenses on inpatient services. Patients' visits to primary healthcare facilities are not increased by health insurance; hospitals remain the primary healthcare provider. Most interstate migrant workers remain unprotected and burdened by the cost of healthcare due to OOP payments in case of medical emergencies. The legal status is a crucial predictor of migrants' access to affordable and adequate health care in a country.

## Introduction and background

The cost of healthcare services people directly pay for is an out-of-pocket expense (OOP) [1]. Unregulated direct charges increasing OOP expenses are a significant barrier to health care. High out-of-pocket medical expenses are often a result of inadequate and a lack of health insurance. Out-of-pocket payments in India account for around 62.6% of overall health expenditure, making it one of the highest in the world [2]. Because of a lack of health insurance, increased expenses, administrative challenges, a lack of public health infrastructure, and limited access to these facilities, migrant workers have difficulty getting healthcare even under normal conditions [3]. Migration is the term for people moving across national or international borders to live somewhere other than where they usually do. India had 45.6 crore migrants in 2011 (38% of the population), up from 31.5 crore migrants in 2001, as per the 2011 Census (31% of the population) [4]. According to official estimates, Indian industries employ about 100 million internal migrant workers, significantly impacting India's economy [5]. Globally, migrant workers numbered 169 million in 2019 [6]. These workers are employed in hazardous occupations. They often face human rights violations, abuse, human trafficking, and violence while working for less pay, longer hours, and in poorer conditions than non-migrants [7].

Out-of-pocket healthcare expenses or payments frequently include the cost of hospital bills, prescription drug costs, and physician consultation fees. Although out-of-pocket payments cover expenses for complementary and alternative treatment, special nutrition and transportation connected to health are not. Out-of-pocket(OOP) payments as a percentage of a household's capacity to pay are what is referred to as the burden of health expenditures [8]. Migrant workers from poor households have fewer resources than wealthy households, so their ability to spend on health care is limited. In the long run, migrant workers may avoid seeking medical attention due to the difference in their out-of-pocket expenditure(OOPE) between doctor visits and the use of over-the-counter (OTC) medications [9]. Free medicines, surgery, and diagnostic tests in public health centers may reduce India's high OOPE and medical poverty [10].

This narrative review aims to gain insight into why migrant workers face out-of-pocket expenditures on health in India. The primary goal is to understand migrant workers' health and what causes them to incur high out-of-pocket (OOP) health expenses. Additionally, it was stressed to explore the inclusion of migrant workers within the current healthcare financing schemes in India. This review will be helpful from the public health perspective in illustrating the need for studies and research on the health rights of migrant workers, their healthcare finances, and the social protection of such vulnerable populations who are poor and marginalized. Focusing on this study area would reduce the financial hardship experienced by migrant workers due to out-of-pocket medical expenses. The existing healthcare policy could be adapted for migrant workers to effectively eliminate health inequity and fulfill the objectives of "health for all" and universal health coverage.

## Review

This review focuses on 'out-of-pocket health expenditure among migrant workers in India'. For the literature search, databases like PubMed and Google Scholar were used to find all original and reviewed articles with original reports. Several keywords and MeSH terms were used interchangeably and in combination to find all relevant articles that included out-of-pocket health expenses of migrant workers. These terms were 'Out-of-pocket expenditure,' 'migrant workers', 'migrant laborers', 'OOPE', 'out-of-pocket health expenditures,' 'out-of-pocket payments, and 'health care financing'. In addition, an online search was conducted using a search engine such as Google to discover government websites and portals to obtain relevant and updated data. The maximum number of articles covered in this review ranges from 2000 to 2022 and are in English with free full text. All types of studies were considered if they were found to be relevant to our review topic. Articles were excluded if they were not relevant to migrant workers' health and out-of-pocket health expenses.

Discussion

OOPE on Health for Migrant Workers in India

Out-of-pocket costs make up a sizable portion of healthcare expenditure in India [11]. The government's healthcare payment methods have been linked to the population's health and economy. While specific payment mechanisms, like social insurance and other prepaid options, have encouraged people to use the official healthcare system, others, such as OOPE, have discouraged people from getting treatment. Additionally, negative coping mechanisms, including borrowing money and selling assets, have been connected to out-of-pocket expenditures [12].

The term "interstate migrants" refers to people who migrate inside the country in pursuit of a living [13]. Interstate migrants, primarily laborers, comprise a sizable portion of India's workforce. Interstate, intra-district, inter-district, and intra-district migrants (including migrant workers) increased from 309.3 million in 2001 to 9.9 million in 2011, with migrants interstate (including migrant workers) increasing from 1.1 million in 2001 to 5.2 million in 2011 (Census, 2011). Since 2011, interstate migration has been reported to have increased annually by approximately 9 million people through 2016 (GoI, 2018). For decades, Bihar and Uttar Pradesh (UP) were the top states for migration, followed by Rajasthan and Odisha. Maharashtra, Delhi, Gujarat, and West Bengal are the most populous states in terms of immigration [14]. 

Out-of-pocket health care expenses (OOP) cover direct and indirect expenses such as consultations, medications, diagnosis, hospital admission fees, transportation, accommodation, and food. These prices are shown in INR [1]. According to the National Health Accounts Estimates for 2016-17 (GoI), inpatient and outpatient care account for most of the current healthcare expenditure (52.4 percent). 

The other significant expenditure category is pharmaceuticals and other medical goods (primarily prescribed medications). This expenditure is dominated by private sector healthcare providers and is primarily funded by households out of their own pockets (63.2%); the government's contribution is significant and mostly non-insurance based. The government plays a critical role in healthcare provision or financing in India. High out-of-pocket expenses combined with limited coverage of contributory and employer-based insurance raise concerns about affordability and equity in healthcare access in the context of the vulnerability of low-income segments of society, such as migrant workers [14].

Determinants of Migrant Workers' General Health

Inadequate insurance coverage and high out-of-pocket (OOP) payments for healthcare place migrant workers, particularly those who are irregular migrants, at risk for serious health issues [15]. The workers' health is compromised due to the numerous occupational and environmental hazards they are exposed to [16]. Various factors influence migrants' health, including travel circumstances, food and nutrition consumption, accessibility of drinking water, and other requirements. Migrants are exposed to health risks at their destination, including communicable diseases and occupational health hazards such as respiratory problems, lung diseases, allergies, kidney and bladder infections, back pain, and malnutrition are some of the most common conditions [17]. Migrants frequently suffer injuries and accidents on the job but are not provided with medical care or compensation. 

Vulnerabilities faced by migrants are poverty, powerlessness, language barriers, discrimination, lack of identity, lack of access to (education, health, livelihood, and food security), inability to access assistance to government programs and services, unsafe working conditions, lack of knowledge about rights, entitlements, and the general application of laws, along with inadequate or nonexistent legal protection, unreliable savings, and remittance facilities [18].

Migrant construction workers are more likely than workers in many other industries to develop certain health disorders and illnesses, particularly occupational injury and fatality [7]. Many workplaces provide certain perks and benefits to their employees for their safety and protection. These facilities include legal protection, written contracts, hygienic living conditions, compliance with health and safety rules and regulations, assurance of medical insurance coverage, and details on whether workers got their compensation benefits. Local governments and businesses should hire migrant workers from authorized recruitment agencies to provide the amenities specified above.

Internal migrant workers are practically highly susceptible to new infections due to interdependent risk factors such as socioeconomic status, chronic malnutrition, occupational hazards, a lack of proper sanitation, unsanitary living conditions in their urban accommodations, and pre-existing respiratory illnesses [8]. Regulatory bodies must improve the working conditions of construction workers by ensuring good living conditions, sanitation facilities, and the availability of personal protective equipment on construction sites [7]. 

Migrant Workers' Barriers to Healthcare Access

The primary healthcare utilization among migrants workers remains low due to a variety of factors, including high costs of private health care, conflicting work schedules and the availability of healthcare practitioners, the cost of missed work hours or days, long distance to access services, and associated transportation issues, apparent disconnection from government healthcare institutions at the destination, and language barriers [17]. Language skills created a cognitive barrier as a result of migration. Due to their limited knowledge of the local languages, some employees needed help understanding information on health and healthcare services [19].

Lack of essential social, legal, and health protection increases workers' vulnerability. Studies have also shown that despite being closer to urban health services than ever before, migrants rarely get access to them. Additionally, they are not allowed to participate in government health services in the destination area just because they are immigrants [20]. Situations like these have a significant impact on the general health and well-being of migrant workers [21]. 

In Mumbai, Pune, Ahmedabad, Delhi, and Bangalore, migrant workers appeared to prefer private health facilities over the free services provided by government hospitals since there was less waiting time. Long waiting times in government hospitals could cost them a day's wage, which they could not afford [22]. As a result of the limited availability of public healthcare, poor migrants frequently seek services from private healthcare providers, resulting in high out-of-pocket health expenses [23].

State and Central Schemes for Migrant Workers

The existence of a few good State and Central programs must be acknowledged. These programs aim to provide social security and health care to migrant workers. However, according to the field survey findings, they haven't received any benefits from these programs. The distribution of the health insurance system among migrant workers is unequal [24]. The ability to benefit from government programs was only available to a small percentage of migrant workers. The range of interstate migrant worker access to programs is 0.5 to 27.5 percent [25]. For example, the reach of AB-PM JAY, i.e., Ayushman Bharat-Pradhan Mantri Jan Arogya Yojana (3.25% in Gujarat, 3.5% in Haryana and 3% in Maharashtra) and NSAP(National Social Assistance Programme) (Delhi 3.75%, Gujarat 4%, Maharashtra 4.25% and Haryana 4.5%) is shockingly low, in contrast, Pradhan Mantri Jan-Dhan Yojana (Delhi-27.25 percent, Gujarat-19.75%, Haryana-22.25%, Maharashtra-23%) has a marginally higher result [25]. The information above is summarized in table 1.

**Table 1:** Interstate migrant workers' access to benefits at the central level Data source [25]

Schemes at the central level	Services/Benefits	Delhi	Gujarat	Haryana	Maharashtra
		Level of reach/Access in percentage			
Ayushman Bharat-Pradhan Mantri Jan Arogya Yojana(AB -PMJAY)	Health insurance scheme. Free medical treatment is offered to eligible patients at empanelled private hospitals.	Operational state program	3.25	3.5	3
NSAP(National Social Assistance Programme)	consists of the National Family Benefit Program, the Annapurna Scheme, the Widow Pension Scheme, the Disability Pension Scheme, and the Old Age Pension Scheme.	3.75	4	4.5	4.25
Pradhan Mantri Jan-Dhan Yojana	The provision of financial services- Banking, credit, remittance, insurance, and pension accounts.	27.5	19.75	22.25	23

Below Poverty Line (BPL) families can access a range of hospital-based medical services through the RSBY health insurance program, launched by the Ministry of Labour and Employment of the GOI in April 2008 [2]. However, the program's reach is limited and does not protect people from paying out-of-pocket for outpatient healthcare [26]. Better information on healthcare financing is necessary for wise policy change in healthcare reform [27]. 

Reasons for High Out-of-Pocket Health Expenses

The severity of the disease was the main factor contributing to the increase in OOP. Undocumented and unskilled migrants, in particular, frequently perform work with limited social protection, have limited access to healthcare and other social services, and are vulnerable to exploitation [28]. Comparing migrant workers (MWs) to the city's permanent citizens, MWs typically earn less money and have poorer sociodemographic profiles [29]. Healthcare provided in hospitals is India's primary emphasis of health insurance programs. Most schemes do not cover the cost of prescription drugs or outpatient care, creating a significant gap in their goal of removing the impoverished associated with health care [30]. Existing health insurance programs provide migrant workers with insufficient protection against out-of-pocket expenses [31].

The migrant community is at a higher risk than any other group, even though their access to healthcare is limited. Most migrant workers who traverse state boundaries go unprotected. Given that workers employed in the informal sector do not have established, documented, long-term employer-employee relations and that their employment is irregular and seasonal, organizing social security schemes is challenging [19]. Health insurance schemes at the local level for workers facilitate financial support. But they could not avail of these benefits because of challenges in registration as local labor welfare board beneficiaries or getting a BPL(Below Poverty Line) status [32]. In addition to being uneducated, unskilled, and from a backward area, migrant workers are not organized under any trade unions, and their labor standards are not covered by the government or trade unions. They do not receive the minimum pay required by the Minimum Wage Act [33]. 

Strategy to Lower Migrant Workers' High Out-of-Pocket Health Care Costs

Whether government or private, most Indian Health insurance schemes are inpatient-focused, meaning they do not often cover a significant portion of outpatient expenses. Therefore, if the government and privately provided insurance schemes become more acceptable and socially relevant, health insurance design must be reviewed [34].

The perception of access to health services by vulnerable populations in India must be assessed [19]. To reduce high OOPE, India must provide migrant workers with adequate and appropriate access to government welfare programs and health care [25]. By improving healthcare service utilization, the role of the host state's Department of Health could be to facilitate the registration process for Health Centres and Government Hospitals. Extending the Employees State Insurance Scheme(ESIS) to unorganized employees and impoverished urban migrants should be addressed, so they are covered by required social protection [35].

Health Insurance Cards (HICS) assisted insured migrants in reducing financial costs associated with OOP at the point of care [36]. A step in the right direction toward providing migrant workers with health care services would be to set up health camps and issue health cards. Health cards containing all their identifying information and medical data may be given to all migrant workers. Under the National Digital Health Mission, every Indian will receive an ID card from the Indian government that will include all relevant information regarding their medical condition [25]. 

Health disparities among migrant workers are a national and international concern. The world's most ambitious dream is to have universal health coverage (UHC) [37]. As stated in SDG target 3.8, achieve universal health coverage (UHC), which includes access to high-quality essential healthcare services, financial risk protection, and access to essential medications and vaccination for everyone [38]. Including migrant workers at all levels of health will reduce further disparities in the healthcare sector. To achieve UHC, low- and middle-income countries need to strengthen their healthcare systems and pay more attention to the larger goal of health equity [15].

## Conclusions

Migrant workers engage in both formal and informal sectors. Their profession encompasses a variety of jobs ranging from construction and brick kiln industry to the agriculture sector and so on. Through contracts with established regulatory organizations, these employees are given jobs. In well-established industries, workers have all advantages and conveniences, including healthcare coverage and insurance. However, these amenities are unavailable to those who work in the unorganized sectors. Given the above, migrant workers, as a vulnerable population, encounter many difficulties while attempting to access the healthcare system for medical care and treatment during routine and emergency visits. Most health insurance plans only pay for inpatient care for serious conditions. While seeing a doctor for routine treatment, migrant workers must pay for outpatient services such as consultation costs, diagnostic tests, and prescription medications. Moreover, only some health plans are transferable between states. Because of these circumstances, migrant workers are burdened with out-of-pocket healthcare expenses. 

They significantly contribute to the country's development and economy, but their health rights and protection are threatened. Regardless of the advantages of migration, socioeconomic factors contribute to health inequities among migrant workers. Because of immigration policies, legal status, and cultural or linguistic barriers, they frequently endure hardship due to their occupation. To strengthen the present healthcare management, further research is needed in healthcare financing for migrant workers in India. More studies and research in this area will help migrant workers cope with their healthcare expenditure burden. Different healthcare policies and insurance schemes exist in India at the state and national levels. Those initiatives being implemented at the central level are not yet being adopted in all Indian states since some states have health programs and policies for migrant workers. The central and state governments should work collaboratively to prevent migrant workers from paying for healthcare out of pocket. Health insurance and healthcare coverage should be transferable between states and the center, as well as across states. NGOs and corporate entities should also step forward to advocate for migrant workers' safety and health and assist in alleviating this healthcare expenditure burden. Migrant workers should be effectively included in all aspects of the existing health system to improve healthcare delivery, administration, and management in a time of need.
